# Academic Possible Selves, Motivational Beliefs, and Self-Regulation Among Adolescents Attending General and Vocational Schools: Does the Type of School Matter?

**DOI:** 10.3390/bs15020158

**Published:** 2025-02-01

**Authors:** Evropi Efthymiadou, Eleftheria N. Gonida, Grigoris Kiosseoglou

**Affiliations:** School of Psychology, Aristotle University of Thessaloniki, GR 54124 Thessaloniki, Greece; gonida@psy.auth.gr (E.N.G.); kios@psy.auth.gr (G.K.)

**Keywords:** academic motivation, academic possible selves, academic self-regulation, adolescents, identity-based motivation theory, secondary education, vocational schools

## Abstract

Academic possible selves (PSs) are defined as future self-representations about education and academic outcomes, recognized for their motivational power, especially in challenging situations. This study aimed to (i) explore the salience of academic PSs among senior high school students, considering sociodemographic factors; (ii) investigate the relationships among perceived contextual factors, PS constructs, difficulty mindsets, and academic outcomes; and (iii) examine differences between general and vocational school students across these variables and their relationships. A sample of 598 10th graders (i) reported their two most important hoped-for and feared PSs as well as their strategies to achieve/avoid them and (ii) completed scales measuring demographics, perceptions about parents’ and teachers’ beliefs, perceived efficacy for attaining PSs, perception of school as a path, difficulty mindsets about academic tasks, academic self-regulation, and achievement. The results indicated that academic PSs were salient among adolescents’ hoped-for PSs, with general school students reporting academic PSs more frequently and showing more positive motivational beliefs. Path analysis indicated the role of the study variables in academic self-regulation and achievement, with school type moderating these effects. The findings emphasize the importance of supporting students’ academic PSs and tailoring context-based interventions to foster academic outcomes in diverse school settings.

## 1. Introduction

During times of global change and uncertainty, adolescents, particularly those from socioeconomically disadvantaged and less supportive environments, are at increased risk of developing negative self-beliefs and lower educational expectations, experiencing poor academic outcomes, devaluing schooling and academic learning, and having limited access to future academic and career opportunities (Gonida & Lemos, 2019; Schoon & Mortimer, 2017; Schoon & Ng-Knight, 2017). This issue is also pertinent to vocational school students, who often come from socioeconomically and academically disadvantaged backgrounds (Kiprianos & Christodoulou, 2015; Van der Veen & Peetsma, 2019) and tend to develop less adaptive motivational and academic profiles (Cents-Boonstra et al., 2019; Chishima & Wilson, 2021; Dubeau et al., 2017). Given that students in vocational schools report low levels of future orientation, goal setting, career planning, and connection between the present and future (Chishima & Wilson, 2021; Dias & Cadime, 2017; Konidari, 2024; Peetsma, 2000), it is important to investigate their future time perspective and its relation to motivation within this student population.

One construct that encompasses the future time dimension and has been linked to academic motivation is the concept of possible selves. Possible selves are cognitive representations of the future desires and fears people have for themselves, reflecting the future aspect of the self-concept. They encompass positive/hoped-for selves, which include self-representations that one wants and aspires to be, and negative/feared selves, which represent negative images people fear becoming and wish to avoid (Markus & Nurius, 1986). Specifically, academic possible selves are the future self-representations about education and academic outcomes. Academic possible selves have been acknowledged as drivers of motivation and self-regulation in academic settings, as they facilitate students to direct attention, regulate and guide behavior, and sustain effort toward achieving their future aspirations and avoiding their future fears in the academic domain (O’Donnell & Oyserman, 2022; Oyserman & Fryberg, 2006). However, further research is needed to examine how the salience of these future self-images within the academic domain differs among students from diverse sociodemographic and educational backgrounds. Additionally, it is important to investigate how these images, when combined with other perceptions, can motivate students in ways that contribute to positive academic outcomes.

The goal of the present study was to investigate adolescents’ academic motivation through the lens of academic possible selves and related motivational self-beliefs, as well as their relationships with academic self-regulation and academic achievement. Moreover, this study examined students attending general and vocational senior high schools to investigate potential differences among the two groups of students. Specifically, the aim of this study was twofold: (a) to explore the salience of the academic domain in adolescents’ future self-descriptions, identifying which factors influence the salience of academic possible selves, and (b) to identify the role of academic possible selves and relevant motivational self-beliefs in academic self-regulation and academic achievement among students in general and vocational senior high schools.

### 1.1. Academic Possible Selves

Academic possible selves refer to future self-representations, both positive and negative, related to education and academic outcomes (Oyserman & Fryberg, 2006). These self-representations have been explored in the possible-self literature in various ways, ranging from those focused on school—including images of oneself as a student (e.g., being an A student or a lazy student)—to those related to broader academic outcomes beyond the school context (e.g., being proficient in the use of technology and digital tools or being unfamiliar with foreign languages) (e.g., Oyserman, 2022). They also differ in temporal focus, ranging from short-term representations, such as next-year possible selves (e.g., getting better grades or not passing the class) (e.g., Bi & Oyserman, 2015; Lee, 2022; Oyserman et al., 2004) to long-term academic self-representations (e.g., envisioning oneself as a university student or not pursuing further studies) (e.g., Leondari & Gonida, 2008; Zhu & Tse, 2016). Another subcategory of academic possible selves includes education-dependent career selves, which represent images within the career domain that involve academic characteristics and require an academic path to achieve them (e.g., becoming a lawyer or not finding a job related to one’s studies) (e.g., Li et al., 2023).

Evidence shows that adolescents report possible selves, aspirations, and long-term goals in the academic and career domains, which they prioritize and value as being important compared to other domains (Knox et al., 2000; Leondari et al., 2009; Leondari & Gonida, 2008; Nurra & Oyserman, 2018; Oyserman et al., 2006; Yau et al., 2021; Yowell, 2000, 2002; Zhu & Tse, 2016). These future self-images, representing educational and occupational success, are salient among adolescents in different cultural and ethnic groups (Chang et al., 2006; Knox et al., 1998; Zhu & Tse, 2016) and among students in more vulnerable and deprived contexts (Bi & Oyserman, 2015; Dumont et al., 2022; Matías-García et al., 2023; Yau et al., 2021). The salience of these themes highlights the developmental aspect of possible selves, indicating that the content of possible selves is related to developmental life tasks and achievements that characterize adolescence, as one of the main developmental tasks during adolescence is the formation of career and academic aspirations and goals, career planning, and the exploration of academic and career paths (Leondari et al., 2009). At the same time, despite the common themes reported in future self-images, differences in the content of possible selves exist among diverse groups of students. This supports the idea that possible selves are constructed within the sociocultural contexts in which adolescents develop (Markus & Nurius, 1986; Oyserman & James, 2009).

### 1.2. The Motivational Power of Academic Possible Selves and Their Links to Self-Regulation and Achievement Through Identity-Based Motivation Theory

Several studies have indicated that academic possible selves are associated with various positive academic outcomes, including better classroom behavior, school persistence, engagement, and school performance (Leondari et al., 1998, 2009; Leondari & Gonida, 2008; O’Donnell & Oyserman, 2022; Oyserman et al., 2002, 2004, 2006). However, evidence suggests that academic possible selves alone are not sufficient for academic self-regulation and success (Oyserman, 2013). Although many students may envision possible selves in the academic domain, they do not always regulate their behavior and learning to attain their hoped-for academic possible selves or avoid their feared academic selves. This challenge is more likely among students in less advantaged contexts, such as low-income and low-education families, ethnic minority groups, high-poverty and high-unemployment areas, and schools with limited resources and support (Oyserman, 2013; Oyserman & Fryberg, 2006; Oyserman & Lewis, 2017). As a result, the literature on possible selves has focused on identifying those motivational features of academic possible selves that may promote students’ academic self-regulation and achievement. An evidence-based framework for understanding when and how possible selves can motivate behavior is provided by Identity-Based Motivation (IBM) theory, a social–cognitive theory of self and self-regulation developed by Daphna Oyserman and her colleagues (Oyserman, 2007, 2015). IBM theory highlights the significance of individuals’ contexts and their social and personal identities (i.e., self-definitions regarding personal traits, social groups, and social roles) in their self-regulation (Elmore & Oyserman, 2012; Oyserman et al., 2017).

#### 1.2.1. Experienced Relevance and Connection Between Current and Possible Self

According to IBM theory, a key condition for possible selves to have motivational power is their perceived connection and relevance to students’ current identities, contexts, and actions (Oyserman et al., 2017). This means that students must see a link between their present identities and possible selves, recognizing their present actions as being important and useful for moving toward their hoped-for possible selves and away from their feared ones. The concepts of connection and relevance are closely related to the idea of self-continuity, which emphasizes the importance of seeing one’s future self as a continuation of the present self (Sedikides et al., 2023). This self-continuity fosters greater engagement in academic and career planning, encouraging students to study more and achieve better academic performance (Chishima & Wilson, 2021).

*Seeing school as a path.* Regarding academic possible selves, one way to establish such a connection is for students to view school as a path to their future. In other words, students need to perceive school, academic tasks, and academic efforts as being relevant and meaningful steps toward their future self. This perception increases the likelihood of students taking action to attain their possible selves, putting effort into academic tasks, and performing better, because they perceive school-focused behaviors as being relevant and connected to their future (Landau et al., 2017; Nurra & Oyserman, 2018).

*Academic Possible Self Plausibility.* When a possible self is perceived as being plausible, it is experienced as more relevant to current choices and more connected to the present (Oyserman & Horowitz, 2023). Plausibility, defined as the extent to which academic possible selves are linked to strategies, reflects the structure of academic possible selves rather than solely their content or centrality (O’Donnell & Oyserman, 2022). Strategies represent the necessary behaviors adolescents should adopt to attain their hoped-for possible selves and avoid their feared ones (Oyserman et al., 2004; Oyserman & James, 2009). In the academic domain, strategies include behaviors such as completing daily homework, paying attention in class, participating in classroom activities, and seeking academic help. Possible selves that include a strategy plan for their attainment or avoidance are considered as plausible (Oyserman et al., 2004; Oyserman & Horowitz, 2023). Plausible academic possible selves have been associated with higher levels of in-class participation and effort in academic tasks (Oyserman et al., 2004), fewer disruptive behaviors in class (Oyserman et al., 2006), and improved academic performance (Horowitz et al., 2020; O’Donnell & Oyserman, 2022; Oyserman et al., 2004). Furthermore, Lee (2022) suggested that in addition to the association between possible selves and academic self-regulatory strategies, students’ commitment to implementing these strategies is critical in predicting higher levels of self-regulation and academic achievement.

*Self-Efficacy.* Perceived efficacy in achieving hoped-for possible selves and avoiding feared ones can also imply relevance, and it can be conceptualized as an aspect of self-regulating possible selves (see Oyserman & Horowitz, 2023), particularly academic possible selves (Frazier et al., 2021). Although this concept has not been extensively studied in the literature on possible selves, studies on future orientation support the idea that adolescents are more likely to plan their academic future if they believe they can influence their future through their actions (Nurmi, 1991, 2005).

#### 1.2.2. Difficulty Mindsets About Academic Tasks

Another core premise of IBM theory is that the interpretation of difficulty experienced toward pursuing possible selves matters for engagement and self-regulation. The theory suggests that difficulty in academic tasks must be interpreted by students as a sign that the task is important and the effort is worthwhile, indicating the significance of achieving their academic possible selves. Studies have shown that when students interpret difficulty in academic tasks as indicating importance, they feel more capable of achieving their academic possible selves. They are more likely to engage and persist in tasks, develop strategies, and achieve higher academic performance. Conversely, interpreting experienced difficulty as a sign of impossibility suggests that the task, and the related possible self, is unattainable. This interpretation reduces the likelihood of putting effort into achieving academic possible selves (Aelenei et al., 2017; Burbidge et al., 2024; Oyserman, 2024; Oyserman et al., 2018; Smith & Oyserman, 2015).

According to IBM theory, these inferences from the experience of difficulty are influenced by the congruence between salient identities and the behaviors required for the attainment of possible selves. Congruence refers to the alignment between students’ social identities and their academic behaviors and achievements. Students whose salient identities include academic achievement are more likely to interpret difficulties in academic tasks as signs of importance rather than as signs of impossibility (Elmore et al., 2016; Oyserman et al., 2018, 2021; Oyserman & Horowitz, 2023). Furthermore, when possible selves are plausible, experienced as being relevant to current choices, and individuals feel efficacious or certain to attain them, difficulties are more likely to be interpreted as signals of importance. Conversely, when possible selves are not plausible, are not seen as being relevant to current choices, and lack connection to efficacy beliefs, difficulties are more likely to be interpreted as signals of impossibility (Oyserman, 2024; Oyserman & Horowitz, 2023). The opposite can also occur, as Burbidge et al. (2024) suggested in their study. Specifically, they suggested bidirectional relationships between difficulty mindsets and students’ certainty of attaining their next-year academic possible selves through a recursive process, where difficulty as importance bolsters students’ certainty of attaining their possible selves and certainty reduces difficulty as impossibility.

### 1.3. The Role of Sociodemographic and Contextual Factors

Contextual factors, such as parental socioeconomic status (SES), parental and teacher support, gender norms, and school context, influence academic self-regulation and school-focused effort, both directly and indirectly, through students’ academic possible selves and motivational beliefs about self and education (e.g., self-efficacy, perception of school as a path to the future, and difficulty mindsets).

Although several studies have found that students report academic-related future self-descriptions despite coming from disadvantaged socioeconomic backgrounds (Bi & Oyserman, 2015; Dumont et al., 2022; Matías-García et al., 2023; Oyserman et al., 2011; Yau et al., 2021), there are studies indicating that students from families with a low socioeconomic status report lower future education orientation (Kerpelman & Mosher, 2004) and fewer long-term educational expectations (e.g., attending or finishing college) (Chang et al., 2006; Schoon & Ng-Knight, 2017) and are less likely to aspire to higher education (Hadjar et al., 2021). Students from lower-socioeconomic (SES) backgrounds report more abstract academic possible selves and lack specific strategies for achieving them (Oyserman et al., 2011). These students also report lower levels of general and academic self-efficacy (Wiederkehr et al., 2015). IBM theory highlights the critical role of SES in shaping constructs such as relevance, connection, and experienced difficulty. Students from lower-SES backgrounds are less likely to view academic achievement as being congruent with their social identities, to perceive connection between their present actions and possible selves, or to see school as a path to their future (e.g., Oyserman, 2013; Oyserman & Horowitz, 2023). Furthermore, they are more likely to interpret experienced difficulty in school as a sign of impossibility rather than importance, as their salient identities may not include academic achievements (Oyserman, 2013; Oyserman & Lewis, 2017).

However, when parents in economically underprivileged families engage in their children’s education, encourage them, and have educational values, students are more likely to develop positive academic possible selves (Carey, 2022; Oyserman et al., 2007). Students from diverse backgrounds perceiving higher levels of parental pragmatic support or support for academic achievement tend to report more academic- and career-related possible selves and have a higher future education orientation (Kerpelman et al., 2008; Zhu et al., 2014). Moreover, when teachers of ethnic minority students support them in viewing education as a feasible option, these students are more likely to envision an academically oriented future (Pizzolato, 2006).

Many studies have found that female students articulate more hoped-for (Hadjar et al., 2021; Kerpelman et al., 2008; Kerpelman & Mosher, 2004; Leondari et al., 2009; Yau et al., 2021; Zhu et al., 2014; Zhu & Tse, 2016) and more feared (Knox et al., 2000; Leondari et al., 2009; Leondari & Gonida, 2008; Oyserman & Fryberg, 2006; Zhu & Tse, 2016) academic future images of themselves compared to males. Other studies have not found differences in frequency, but they have found that girls prioritize their academic possible selves and future goals (Chang et al., 2006; Yowell, 2000). Others did not find any significant gender differences regarding the salience of the academic domain in students’ projections of their selves (Li et al., 2023; Matías-García et al., 2023). Several studies suggest that females, compared to males, report more strategies for achieving or avoiding their academic possible selves (Konidari, 2021; Oyserman et al., 2011; Zhu et al., 2014; Zhu & Tse, 2016), as well as exert greater effort to attain their educational expectations (Schoon & Ng-Knight, 2017). Girls have been found to be more optimistic when envisioning negative future selves (Leondari et al., 1998), while other studies did not find gender differences in the perceived likelihood of achieving or avoiding possible selves (Knox et al., 1998; Zhu & Tse, 2016).

Research has also shown that gender interacts with SES. Studies indicate that boys are more vulnerable than girls to the negative effects of disadvantaged backgrounds. Specifically, Oyserman et al. (2011) found that boys from lower-SES backgrounds reported fewer strategies for achieving their academic possible selves compared to girls. Additionally, Li et al. (2023) found that boys from lower-SES backgrounds reported lower perceived likelihoods of achieving their career possible selves, a difference not observed among students from higher-SES backgrounds. Schoon and Ng-Knight (2017) found that although boys, regardless of SES, perceive a lower likelihood of attending university and make less school-focused effort than girls, boys from higher-SES backgrounds were more likely to perceive university attendance as attainable and exert greater effort compared to boys from lower-SES backgrounds.

Studies conducted in different cultural environments and investigating the school context have indicated that students attending general schools report more possible selves in the academic domain, while vocational school students report more possible selves in the career domain (Koul et al., 2017; Soidet et al., 2022), with the three most popular hoped-for categories among vocational school students being work related (Konidari, 2021). Moreover, students in vocation-oriented schools report lower future self-continuity and less career planning than those attending academic-oriented high schools (Chishima & Wilson, 2021). Qualitative studies of vocational school students in different countries have also found that most of them have difficulty in describing a detailed future self-image, including a specific path and detailed strategies for attaining that future self (Konidari, 2024). Van der Veen and Peetsma (2019) highlighted the critical role of teachers in vocational schools, noting that students with positive relationships with their teachers exhibited a greater tendency to prioritize school-related tasks over other attractive activities.

### 1.4. The Present Study

Despite a substantial body of research examining the content and motivational power of academic possible selves, a notable gap remains in the possible-self literature. Although the experimental literature has extensively documented the effects of possible selves as well as IBM theory constructs, there is limited research examining the interrelationships among these variables and their contextual predictors and academic outcomes. Specifically, more research is needed on the role of teachers’ and parents’ beliefs on school value and how students’ perceptions about them may predict academic possible self plausibility, self-efficacy, school as a path perception, and difficulty mindsets, and, in turn, how these factors predict academic outcomes, like self-regulation and achievement. Moreover, there is a lack of research examining differences between students attending general and vocational schools regarding the salience of academic future self-representations as well as the potential moderating role of the school type in the pattern of interrelationships among the above variables.

The present study, drawing from IBM theory and evidence supporting that specific aspects of possible selves (e.g., plausibility and efficacy) matter for students’ academic outcomes, was conducted among 10th grade students attending general and vocational high schools. This age represents a developmental period during which future planning, particularly in relation to adult life, assumes greater significance than it did during early adolescence. Additionally, 10th grade is an important year in the Greek educational system (it is the 1st grade of senior high school, both general and vocational), as it marks the point where students, both in general and vocational education, make critical decisions regarding their educational and professional paths, which are closely linked to school curricula. The 10th grade serves as a preparatory year during which students can further explore their interests and career goals, before selecting an academic or vocational specialization for the 11th grade.

The objectives and hypotheses of this study were guided by prior research and were as follows:

*Objectives.* To examine the roles of the sociodemographic background and school type in (a) the salience of academic possible selves and (b) perceived parents’ and teachers’ beliefs about school value, academic possible self plausibility, perceived efficacy for attaining possible selves, perception of school as a path, difficulty mindsets, academic self-regulation, and school achievement;

To investigate the pattern of relationships among perceived contextual factors (i.e., perceptions of parents’ and teachers’ beliefs regarding school value), possible selves (i.e., plausibility, perceived efficacy, and perception of school as a path to the future), difficulty mindsets (difficulty as importance and difficulty as impossibility), and academic outcomes (self-regulation and school achievement);

To examine whether the school type (general or vocational) moderates the relationships among the above variables.

*Hypotheses.* Students with different sociodemographic backgrounds are expected to differ regarding the study variables. Specifically, students in general schools compared to students in vocational schools (Chishima & Wilson, 2021; Konidari, 2024; Koul et al., 2017; Soidet et al., 2022), girls compared to boys (e.g., Hadjar et al., 2021; Kerpelman et al., 2008; Knox et al., 2000; Leondari & Gonida, 2008; Leondari et al., 2009; Oyserman et al., 2011; Yau et al., 2021; Zhu et al., 2014), and students whose parents have higher educational levels compared to those with lower-educated parents (e.g., Hadjar et al., 2021; Oyserman et al., 2011; Oyserman & Lewis, 2017; Schoon & Ng-Knight, 2017; Wiederkehr et al., 2015) are expected to report (a) a greater salience of academic possible selves (Hypothesis 1a), and (b) a more adaptive profile regarding the study variables (Hypothesis 1b).

**Figure 1:**
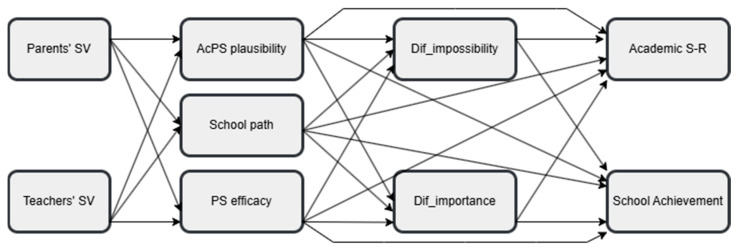
Theoretical model. Notes: Parents’ SV = perceived parental school value for the future; Teachers’ SV = perceived teacher’s school value for the future; AcPS plausibility = plausibility of academic possible selves; School path = perception of school as a path to the future; PS efficacy = perceived efficacy for attaining possible selves; Dif_impossibility = interpretation of difficulty in academic tasks as impossibility; Dif_importance = interpretation of difficulty in academic tasks as importance; Academic S-R = academic self-regulation; School achievement = GPA.

(a) Perceived parents’ and teachers’ beliefs about school value are expected to predict perceived efficacy for attaining possible selves, academic possible self plausibility, and perception of school as a path to the future, based on studies indicating the roles of parents’ and teachers’ beliefs and support (Kerpelman et al., 2008; Oyserman et al., 2007; Pizzolato, 2006; Zhu et al., 2014) (Hypothesis 2a); (b) beliefs about possible selves (efficacy, plausibility, and school as a path) are expected to positively predict a difficulty-as-importance mindset and negatively predict a difficulty-as-impossibility mindset (see Oyserman & Horowitz, 2023) (Hypothesis 2b); (c) beliefs about possible selves are expected to predict academic self-regulation and school achievement (see Horowitz et al., 2020; O’Donnell & Oyserman, 2022; Oyserman et al., 2004 for plausibility; see Oyserman & Horowitz, 2023; Frazier et al., 2021 for self-efficacy; see Landau et al., 2017; Nurra & Oyserman, 2018 for school as a path); (d) difficulty mindsets are expected to predict academic self-regulation and school achievement (Aelenei et al., 2017; Smith & Oyserman, 2015) (Hypothesis 2d). Figure 1 presents our theoretical model.

Based on the differences between the school contexts (e.g., Cents-Boonstra et al., 2019; Chishima & Wilson, 2021), the above relationships are expected to be different between general and vocational school students, with school type serving as a moderator for path relationships (Hypothesis 3a).

## 2. Materials and Methods

### 2.1. Participants

The sample consisted of 598 10th grade students (60.2% female, N = 360) from 12 general schools (388 students) and 10 vocational schools (210 students) located in northern Greece. Among these students, 66% had lived in urban regions for most of their lives, while the remaining 34% had lived in rural regions. Additionally, 13% of the participants had an immigrant background and were bilingual.

Vocational and general schools differed regarding some background variables of the students. Most vocational school students came from more rural (61.7%) than urban regions (38.3%), while the opposite was true for general school students (urban: 80.6%, rural: 19.4%). Parents of general school students had a higher educational level compared to parents of vocational school students. A percentage of 59% of the general school students’ mothers and 45% of their fathers, while 41% of the vocational school students’ mothers and 29% of their fathers had a higher-educational-level degree (e.g., bachelors or masters). Regarding the immigrant background, 14.3% of the vocational school students and 11.9% of the general school students had an immigrant background, and they were bilingual.

### 2.2. Procedure

This study was approved by the Ethics Committee of Aristotle University of Thessaloniki (identification code: 35618/2021; date of approval: 10 February 2021). A pilot study was conducted to assess the psychometric properties of the survey instruments. Access to schools was granted following special permission from the National Institute of Educational Policy, under the Ministry of Education. Schools were contacted, and agreement for participation was obtained from school principals and teaching staff. Written parental consent was secured for all the participating students through signed informed consent forms, which included the details of the study. All the students gave their informed consent for inclusion before they participated. They participated voluntarily after being informed about the aims of the study, their right to withdraw at any point in the procedure, and the assurance of anonymity for the data they would provide. Students completed self-report scales via an online survey that was administered in their schools during one school hour. The completion required approximately 25 min.

### 2.3. Measures

#### 2.3.1. Demographic and Family Background 

Students were asked to provide their gender and whether they attended a general or vocational high school. An open-ended question was used to determine the primary language spoken at home, allowing for the assessment of nationality, immigration background, and bilingualism. Students were also asked to specify the location where they had lived for most of their lives, which was categorized as either urban or rural. As for parental education, the educational levels of the students’ mothers and fathers were assessed. Educational levels were then categorized as either low (ranging from primary school to completing a program at a post-secondary non-university educational institution) or high (ranging from graduating from college/university to pursuing further studies, such as a master’s or doctoral degree).

#### 2.3.2. Possible Selves and Strategies 

Students reported their possible selves and strategies by completing an adapted form of the open-ended Possible Selves Questionnaire (Oyserman & Markus, 1990; Oyserman et al., 1995). Initially, students were provided with a brief explanation of the concept of possible selves. They were then asked to report their two most important hoped-for selves (“Write 2 selves that you would like and hope to become in the future”) and their two feared selves (“Write 2 selves that you fear or wouldn’t want to become in the future”). Following this, participants were asked to describe any strategies they use to achieve or avoid these possible selves (“Are you doing anything during this period to achieve/avoid each self? If yes, what are you doing now?”). An adaptation of the original questionnaire was that we did not specify a particular time frame (e.g., next-year possible selves). This approach, which has been used in previous studies (e.g., Leondari & Gonida, 2008; Leondari et al., 1998), allows participants to generate spontaneously their possible selves and envision them at any point in the future.

Possible selves. Students’ responses for their possible selves were coded using content analysis and following the coding system suggested by Oyserman (2022) with some adaptations. Drawing from previous categorization systems (e.g., Leondari & Gonida, 2008) and aligning with the domains relevant to adolescence (Oyserman & Markus, 1990), we categorized reported possible hoped-for and feared selves into six categories: personal (e.g., open-minded/coward), interpersonal (e.g., parent/being without friends), material/lifestyle (e.g., owner of a big house/homeless), health/physical (e.g., athletic/unhealthy), career (e.g., having a permanent job/unemployed), and academic. The academic category included two subcategories: academic-achievement-focused possible selves (e.g., university student/not attending university) and career–academic possible selves, which relate to jobs requiring studies and an academic path (e.g., architect/not having a job in a field related to my studies). For feared possible selves, an additional category was included: off-track self, encompassing risky or delinquent behaviors (e.g., drug addict or criminal). All the possible selves were double-coded by two independent coders. The interrater reliability was 97% for the hoped-for possible selves and 97.5% for the feared possible selves.

Strategies. Students’ responses for strategies linked to academic possible selves (academic-achievement-focused and career–academic) were coded as either relevant or not relevant for attaining or avoiding these possible selves. Relevant strategies were identified through content analysis and included the following types: (i) academic-school-focused strategies (e.g., studying daily, completing homework, and staying focused during lessons), (ii) motivational–affective strategies (e.g., attending seminars related to future studies and reading books about desired jobs), (iii) non-school-focused strategies required for academic possible selves (e.g., training to pass physical exams required for admission to a police academy), (iv) behavioral self-regulation non-school-focused strategies (e.g., maintaining a healthy sleep pattern and diet and saving money for future studies), and (v) informational strategies (e.g., researching universities that offer the desired field of study).

Academic possible self plausibility. The plausibility of academic possible selves was scored on a 7-point scale (0–6) by assessing academic hoped-for and feared possible selves along with their connected strategies. This evaluation was based on the scoring system suggested by Oyserman (2022), which was adapted to our data. Each point on the scale corresponded to the number of possible selves and strategies reported in the academic domain, with lower scores indicating a combination of fewer academic possible selves and strategies and higher scores indicating a combination of more academic possible selves and strategies. A score of 0 was assigned to students who did not report any possible selves or strategies in the academic domain, while a score of 6 was assigned to those who reported four possible selves in the academic domain and at least four related strategies. The coding system we used is provided in Table 1.

#### 2.3.3. Perceived Efficacy of Attaining Possible Selves

The Academic Efficacy Scale from the Patterns of Adaptive Learning Scales (PALS, Midgley et al., 2000) was appropriately modified to examine the perceived efficacy for attaining possible selves. This modified scale consisted of 6 items on a 5-point Likert scale (e.g., “I am certain I can master the necessary skills to become the person I want to be in the future”). The validity of this scale was tested through exploratory factor analysis (EFA) using principal axis factoring. EFA revealed one factor explaining 46.87% of the total variance. Loadings ranged from 0.62 to 0.75, and Cronbach’s alpha value was 0.84.

#### 2.3.4. Perception of School as Path to Attain Possible Selves

Perception of school as a path to attain possible selves was assessed using a 5-point Likert scale, which assesses the extent to which students believe school is the way to achieve a desired future. The scale was developed by Nurra and Oyserman (2018) and includes 5 items (e.g., “What I’m doing in school is important because it will help me to become what I want to become when I will be an adult”). EFA identified a single factor, accounting for 61.13% of the total variance, with item loadings ranging from 0.73 to 0.83. Cronbach’s alpha reliability value was *α* = 0.89.

#### 2.3.5. Difficulty Mindsets About Academic Tasks

Difficulty mindsets were measured using the Interpretation of Difficulty Scale developed by Oyserman et al. (2015). This scale comprises two subscales, each with 6 items rated on a 5-point Likert scale: Difficulty implies that an academic task is “for me”—difficulty as importance (e.g., “When I’m working on a school task that feels difficult, it means that the task is important”) and difficulty implies that an academic task is “not for me”—difficulty as impossibility (e.g., “When I feel stuck on a school task, it’s a sign that my effort is better spent elsewhere”). EFA identified two factors. Difficulty as importance accounted for 27.50%, and difficulty as impossibility accounted for 23.38%. Because of the multifactorial nature of this scale, we also conducted a confirmatory factor analysis (CFA) to evaluate the fit of the hypothesized two-factor model. The results were fully consistent with those of the EFA. The factorial model fit indices indicated an acceptable fit: *χ*^2^(52) = 130.86, *p* < 0.001, CFI = 0.972, TLI = 0.964, RMSEA = 0.051 with 90% C.I.s from 0.040 to 0.062, and SRMR = 0.032. All the factor loadings were statistically significant (*p* < 0.001), ranging from 0.645 to 0.795 for difficulty as importance and from 0.435 to 0.785 for difficulty as impossibility. The Cronbach reliability values were *α* = 0.88 for the difficulty-as-importance subscale and *α* = 0.83 for the difficulty-as-impossibility subscale.

#### 2.3.6. Perceptions of Parents’ and Teachers’ Beliefs About School Value for the Future

To measure students’ perceptions of their parents’ and teachers’ beliefs about the value of school for their futures, the “Measure of School as a Path” scale (Nurra & Oyserman, 2018) was modified. The modified scale included 5 items tailored for parents (e.g., “My parents believe that what I’m doing in school is important because it will help me to become what I want to become when I will be an adult”) and 5 items for teachers (e.g., “My teachers believe that putting a lot into school activities is important because it will allow me to be the adult I would like to be”). EFA applied separately to each scale indicated a single factor for each scale, explaining 55.68% of the variance for the perceptions of parents’ beliefs scale and 47.59% for the perceptions of teachers’ beliefs scale. Item loadings ranged from 0.70 to 0.77 (parents’ scale) and from 0.61 to 0.75 (teachers’ scale). The Cronbach reliability values were *α* = 0.86 for parents’ beliefs and *α* = 0.82 for teacher’s beliefs.

#### 2.3.7. Academic Self-Regulation and School Achievement

Academic self-regulation was measured using a shortened version of the Self-Regulation Strategy Inventory—Self Report (SRSI-SR), a self-report measure of students’ use of specific self-regulation strategies, developed by Cleary (2006). The shortened version, consisting of 9 items (e.g., “I make a schedule to help me organize my study time”), was derived from the pilot study, in which the scale’s psychometric properties were assessed. EFA conducted in the present study revealed that all 9 items loaded onto a single factor, explaining 38.55% of the total variance, with loadings between 0.5 and 0.73. The Cronbach reliability value was *α* = 0.84. School achievement was measured by asking students to report their 9th grade GPA.

## 3. Results

### 3.1. The Role of Sociodemographic Variables

#### 3.1.1. Sociodemographic Factors and the Salience of Academic Possible Selves

Overall, 70.10% (N = 272) of the general school students and 48.57% (N = 102) of the vocational school students reported at least one academic possible self, either as their first or second self and either as hoped for or feared. To further explore the salience of academic possible selves concerning demographic variables and, specifically, the differences in academic possible selves in relation to the school type, gender, and parents’ educations, a series of chi-squared tests of independence were carried out using adjusted standardized residuals (>1.96 values).

The chi-squared analyses showed a statistically significant association between academic possible selves (i.e., academic-achievement-focused possible selves and career–academic possible selves) and the sociodemographic variables of the school type, gender, and father’s educational level. Specifically, general school students were more likely to report an academic first hoped-for possible self (49.7%), *χ*^2^(1, N = 598) = 19.611, *p* < 0.001, an academic second hoped-for possible self (30.9%), *χ*^2^(1, N = 598) = 13.428, *p* < 0.001, and an academic second feared possible self (10.1%), *χ*^2^(1, N = 598) = 10.135, *p* = 0.001, than vocational school students (31% for the first hoped-for self, 17.1% for the second hoped-for self, and 2.9% for the second feared self). Additionally, girls were significantly more likely to report an academic first hoped-for possible self (47.5%) than boys (36.6%), *χ*^2^(1, N = 598) = 6.998, *p* < 0.01, and they also reported an academic first feared self more frequently (16.7%) than boys (10.9%), *χ*^2^(1, N = 598) = 3.837, *p* = 0.05. Furthermore, students whose fathers had a higher educational level were more likely to report an academic first hoped-for possible self (51.3%) compared to those whose fathers had a lower educational level (38.3%), *χ*^2^(1, N = 583) = 9.560, *p* < 0.01. The mother’s educational level was not statistically significantly associated with the salience of academic possible selves.

#### 3.1.2. The Role of Sociodemographic Factors in Study Variables

A series of one-way MANOVAs was conducted to investigate the multivariate effects of the school type, gender, and father’s and mother’s educational levels on the battery of the nine dependent variables. Next, based on the results of the nine univariate ANOVAs for each independent variable, with Bonferroni correction applied (the alpha value for each ANOVA = 0.05/9 = 0.0056), significant effects of sociodemographic variables were observed (*p* < 0.0056).

The multivariate effect of the father’s educational level was found to be significant: Pillai’s trace = 0.069, *F*(9, 550) = 4.390, *p* < 0.001, observed power = 0.999, with students whose fathers had a higher educational level reporting higher academic achievement, *F*(1, 558) = 19.422, *p* < 0.001, η_p_^2^ = 0.045 (higher-educated fathers: *M* = 17.01/20, lower-educated fathers: *M* = 15.91/20). The multivariate effect of the mother’s educational level was also significant: Pillai’s trace = 0.070, *F*(9, 550) = 4.597, *p* < 0.001, observed power = 0.999. Students with higher-educated mothers reported higher perceived efficacy for attaining their possible selves, *F*(1, 558) = 9.646, *p* = 0.002, η_p_^2^ = 0.017 (higher-educated mothers: *M* = 3.94, lower-educated mothers: *M* = 3.76), and higher academic achievement, *F*(1, 558) = 30.698, *p* < 0.001, η_p_^2^ = 0.052 (higher-educated mothers: *M* = 16.87/20, lower-educated mothers: *M* = 15.69/20).

A significant multivariate effect of gender was also identified: Pillai’s trace = 0.106, *F*(9, 565) = 7.479, *p* < 0.001, observed power = 1.000. Specifically, girls reported higher levels in perception of teachers’ school value beliefs, *F*(1, 573) = 22.747, *p* < 0.001, η_p_^2^ = 0.038 (girls: *M* = 4.34, boys: *M* = 4.07), possible self plausibility, *F*(1, 573) = 10.902, *p* = 0.001, η_p_^2^ = 0.019 (girls: *M* = 2.17, boys: *M* = 1.76), self-regulation, *F*(1, 573) = 17.024, *p* < 0.001, η_p_^2^ = 0.029 (girls: *M* = 3.21, boys: *M* = 2.90), and academic achievement, *F*(1, 573) = 15.336, *p* < 0.001, η_p_^2^ = 0.026 (girls: *M* = 16.68, boys: *M* = 15.84).

The multivariate effect of the school type was found to be significant: Pillai’s trace = 0.300, *F*(9, 565) = 26.915, *p* < 0.001, observed power = 1.000. Univariate ANOVAs with Bonferroni correction indicated the significant effect of the school type (p < 0.0056) on the following variables: perception of parents’ beliefs about school value, *F*(1, 573) = 11.092, *p* < 0.001, η_p_^2^ = 0.019, perception of school as a path, *F*(1, 573) = 8.514, *p* = 0.004, η_p_^2^ = 0.015, possible self plausibility, *F*(1, 573) = 41.708, *p* < 0.001, η_p_^2^ = 0.068, difficulty as impossibility, *F*(1, 573) = 8.474, *p* = 0.004, η_p_^2^ = 0.015, academic self-regulation, *F*(1, 573) = 27.344, *p* < 0.001, η_p_^2^ = 0.046, and academic achievement, *F*(1, 573) = 197.071, *p* < 0.001, η_p_^2^ = 0.256. General school students reported higher values than vocational school students in the perception of parents’ school value beliefs (general: *M* = 4.08, vocational: *M* = 3.84), perception of school as a path (general: *M* = 3.50, vocational: *M* = 3.25), academic possible self plausibility (general: *M* = 2.29, vocational: *M* = 1.49), self-regulation (general: *M* = 3.22, vocational: *M* = 2.83), and achievement (general: *M* = 17.29, vocational: *M* = 14.59), and lower means in interpreting difficulty as impossibility (general: *M* = 2.56, vocational: *M* = 2.78).

Interpretation of difficulty as implying importance did not differ significantly between any of the groups. Overall, students reported moderate levels of difficulty as importance (*M* = 2.95).

### 3.2. Relationships Among the Variables Under Examination

Table 2 presents the correlations among the study variables for both general and vocational school students. These results highlight some similarities and differences in the relationships among these variables across the two school types.

Next, path analysis using Mplus 6 was used (Muthén & Muthén, 1998–2010) to examine the direct and indirect relationships among the study variables (perceived parents’ and teachers’ beliefs, possible-self constructs, difficulty mindsets, academic self-regulation, and school achievement). The variables of the students’ gender and parental educational levels were excluded from the path analysis because of their small effect sizes observed in the MANOVA results presented above.

**Figure 2:**
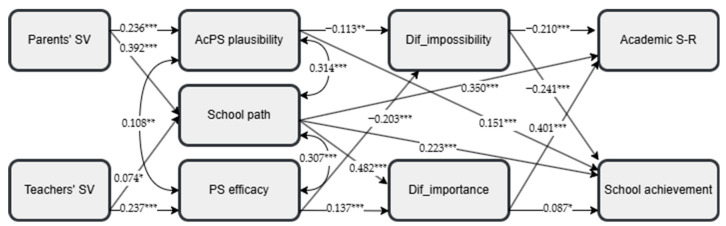
Path model for the total sample. Notes: Parents’ SV = perceived parental school value for the future; Teachers’ SV = perceived teachers’ school value for the future; AcPS plausibility = plausibility of academic possible selves; School path = perception of school as a path to the future; PS efficacy = perceived efficacy for attaining possible selves; Dif_impossibility = interpretation of difficulty in academic tasks as impossibility; Dif_importance = interpretation of difficulty in academic tasks as importance; Academic S-R = academic self-regulation; School achievement = GPA. All the coefficients are standardized; * *p* < 0.05; ** *p* < 0.01; *** *p* < 0.001.

Path analysis was initially conducted on the total student sample, specifying all the potential direct effects. After conducting this preliminary analysis, only statistically significant paths were retained, resulting in a good model fit (Kline, 2011): *χ*^2^(16) = 35.474, *p* < 0.01, *χ*^2^*/df =* 2.217, CFI = 0.983, TLI = 0.962, RMSEA = 0.046 with 90% C.I.s from 0.025 to 0.066, SRMR = 0.024. Figure 2 presents the model and the standardized path coefficients for the total sample. This process was also replicated across the two student groups—general and vocational school students—to test the model fit within each group. Both groups showed a satisfactory model fit. For general school students, the model fit was satisfactory: *χ*^2^(15) = 24.791, *p* = 0.052, CFI = 0.984, TLI = 0.963, RMSEA = 0.041 with 90% C.I.s from 0.000 to 0.069, SRMR = 0.027. The model also fit well for vocational school students: *χ*^2^(17) = 24.653, *p* = 0.102, CFI = 0.984, TLI = 0.966, RMSEA = 0.047 with 90% C.I.s from 0.000 to 0.085, SRMR = 0.046.

The model of the total sample explained 50.5% of the variance in academic self-regulation (*R*^2^ = 0.505, *p* < 0.001) and 21.4% of the variance in school achievement (*R*^2^ = 0.214, *p* < 0.001). For general school students, the model explained 44.9% of the variance in students’ academic self-regulation (*R*^2^ = 0.449, *p* < 0.001) and 23% of the variance in their school achievement (*R*^2^ = 0.230, *p* < 0.001). For vocational school students, 60.9% of the variance in students’ academic self-regulation (*R*^2^ = 0.609, *p* < 0.001) and 16.2% of the variance in school achievement (*R*^2^ = 0.162, *p* < 0.001) were explained by the model variables.

**Figure 3:**
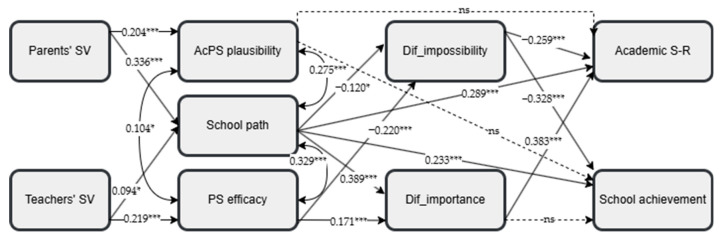
Path model for general school students. Notes: Parents’ SV = perceived parental school value for the future; Teachers’ SV = perceived teacher school value for the future; AcPS plausibility = plausibility of academic possible selves; School path = perception of school as a path to the future; PS efficacy = perceived efficacy for attaining possible selves; Dif_impossibility = interpretation of difficulty in academic tasks as impossibility; Dif_importance = interpretation of difficulty in academic tasks as importance; Academic S-R = academic self-regulation; School achievement = GPA. All the coefficients are standardized; * *p* < 0.05; *** *p* < 0.001; ns = non-significant.

In the general school students (see Figure 3), parents’ perceived school value positively predicted academic possible self plausibility (*β* = 0.204, *p* < 0.001) and the perception of school as a path (*β* = 0.336, *p* < 0.001), while the teachers’ beliefs about school value positively predicted perceived efficacy for attaining possible selves (*β* = 0.219, *p* < 0.001) and the perception of school as a path, albeit with a low beta value (*β* = 0.094, *p* < 0.05). Perceived efficacy for attaining possible selves predicted both difficulty as importance (*β* = 0.171, *p* < 0.001) and difficulty as impossibility (*β* = −0.220, *p* < 0.001). Similarly, the perception of school as a path predicted difficulty as impossibility (β = −0.120, *p* < 0.05) and difficulty as importance (*β* = 0.389, *p* < 0.001). School as a path also predicted academic self-regulation (*β* = 0.289, *p* < 0.001) and school achievement (*β* = 0.233, *p* < 0.001). In this group, academic possible self plausibility did not predict any other variables. Difficulty as importance showed a significant positive effect only on academic self-regulation (*β* = 0.383, *p* < 0.001), while difficulty as impossibility negatively predicted self-regulation (*β* = −0.259, *p* < 0.001) and school achievement (*β* = −0.328, *p* < 0.001).

**Figure 4:**
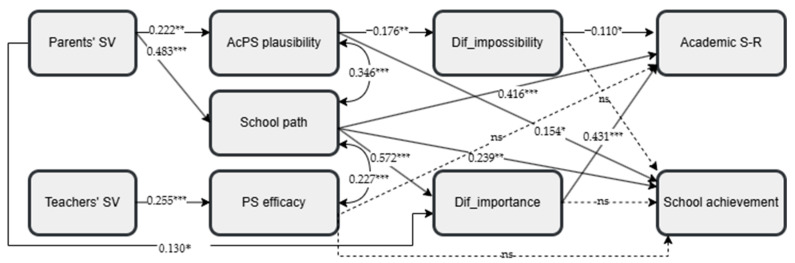
Path model for vocational school students. Notes: Parents’ SV = perceived parental school value for the future; Teachers’ SV = perceived teacher school value for the future; AcPS plausibility = plausibility of academic possible selves; School path = perception of school as a path to the future; PS efficacy = perceived efficacy for attaining possible selves; Dif_impossibility = interpretation of difficulty in academic tasks as impossibility; Dif_importance = interpretation of difficulty in academic tasks as importance; Academic S-R = academic self-regulation; School achievement = GPA. All the coefficients are standardized; * *p* < 0.05; ** *p* < 0.01; *** *p* < 0.001; ns = non-significant.

The model for vocational school students shared similarities with the model for general school students but also revealed some different paths (see Figure 4). Parents’ perceived school value was a predictor of academic possible self plausibility (β = 0.222, *p* < 0.01), the perception of school as a path (*β* = 0.483, *p* < 0.001), and difficulty as importance (*β* = 0.130, *p* < 0.05), while teachers’ perceived school value significantly predicted perceived efficacy for attaining possible selves (*β* = 0.255, *p* < 0.001). Academic possible self plausibility negatively predicted difficulty as impossibility (*β* = −0.176 *p* < 0.01) and showed a positive direct effect on school achievement (*β* = 0.154, *p* < 0.05). The perception of school as a path also significantly predicted difficulty as importance (*β* = 0.572, *p* < 0.001) and directly predicted both academic self-regulation (*β* = 0.416, *p* < 0.001) and school achievement (*β* = 0.239, *p* < 0.01). In this group, perceived efficacy for attaining possible selves did not predict any other variables. Difficulty as importance (*β* = 0.431, *p* < 0.001), and difficulty as impossibility (*β* = −0.110, *p* < 0.05) were significant predictors of academic self-regulation but not of academic achievement.

*Indirect effects.* The statistical significance of the indirect effects was estimated using 5000 bootstrap samples with bias-corrected bootstrap confidence intervals [BC 95% C.I.s]. Table 3 presents all the indirect effects. The analysis showed that perceived parental school value predicted academic self-regulation and achievement through the perception of school as a path in both the general and vocational student groups. Perceived parental school value also predicted difficulty as importance through the perception of school as a path in both groups but difficulty as impossibility through the perception of school as a path among general students and through academic possible self plausibility among vocational students. Moreover, among general school students, perceived teachers’ school value predicted academic self-regulation through perceived efficacy for attaining possible selves, difficulty as impossibility, and difficulty as importance, as well as achievement through perceived efficacy and difficulty as impossibility. Furthermore, perceived teachers’ school value predicted both difficulty mindsets through perceived efficacy for attaining possible selves. Additionally, the perception of school as a path predicted academic self-regulation through both difficulty mindsets among general students, whereas it predicted academic self-regulation only through difficulty as importance among vocational students. Finally, among general school students, perceived efficacy for attaining possible selves predicted academic self-regulation through both difficulty mindsets and academic performance through difficulty as impossibility.

*Multigroup Analysis.* To examine the possible moderation effect of the school type (general vs. vocational), multigroup analysis was conducted. For this purpose, we specified identical paths in each model group, defining the paths that had been found to be significant in at least one group. Both models demonstrated a good fit: for general school students, *χ*^2^(16) = 28.520, *p* < 0.05, *χ*^2^*/df =* 1.782, CFI = 0.980, TLI = 0.955, RMSEA = 0.045, SRMR = 0.034; for vocational school students, *χ*^2^(16) = 22.409, *p* = 0.130, CFI = 0.986, TLI = 0.970, RMSEA = 0.044, SRMR = 0.034.

The unconstrained multigroup analysis model was then compared to a multigroup analysis model with equality constraints on all path coefficients via a chi-squared difference test. The chi-squared difference test (Δ*χ*^2^ = 32.104, Δ*df* = 15) was significant (*p* < 0.01), indicating that overall, the path coefficients differ significantly between general and vocational school students.

Subsequent chi-squared difference tests for each path coefficient showed significant differences in three of them between the school groups. The effect of the difficulty as impossibility on academic self-regulation was stronger in the general school student group (*β* = −0.259, *p* < 0.001) than in the vocational student group (*β* = −0.108, *p* = 0.014; Δ*χ*^2^ = 6.288, Δ*df* = 1, *p* < 0.05). Similarly, difficulty as impossibility predicted school achievement only in the general school group (*β* = −0.328, *p* < 0.001) and not in the vocational school group (*β* = −0.087, *p* = 0.180; Δ*χ*^2^ = 7.019, Δ*df* = 1, *p* < 0.01). Finally, the effect of the perception of school as a path on difficulty as importance was positive in both groups but stronger for vocational students (*β* = 0.607, *p* < 0.001) than for general students (*β* = 0.390, *p* < 0.001; Δ*χ*^2^ = 7.895, Δ*df* = 1, *p* < 0.01).

## 4. Discussion

The aims of the present study were to examine the role of the sociodemographic background and school type in shaping the salience of academic possible selves, perceived contextual factors, aspects of possible selves, difficulty mindsets, and academic outcomes while also investigating the pattern of relationships among these variables and assessing whether the school type moderates these relationships. The results highlighted the salience of the academic domain in adolescents’ future self-concepts, particularly among general school students, while sociodemographic factors, including parental education and gender, were found to play a role in the salience of academic possible selves, plausibility, and perceived efficacy, as well as academic outcomes. The school type was also found to be a significant factor, indicating a more positive profile for general school students regarding the study variables. Path analysis revealed that perceived parents’ and teachers’ beliefs, aspects of possible selves, and difficulty mindsets significantly predicted academic self-regulation and achievement, with the school type moderating these relationships.

### 4.1. The Role of Sociodemographic Variables

Academic possible selves were found to be the most frequently reported domain among hoped-for possible selves for general school students and the second most frequent for vocational school students. Additionally, more than half of general school students and nearly half of vocational school students reported at least one academic possible self as either hoped for or feared. These findings highlight the salience of this domain in adolescents’ self-concept, especially among general school students. These academic possible selves included both academic-achievement-focused possible selves (e.g., university student/not attending university) and career–academic possible selves (i.e., self-representations related to jobs requiring studies and an academic path).

Regarding the role of the parental educational level, our findings indicated that students whose fathers had higher levels of education reported more academic possible selves, particularly hoped-for selves, while students with more highly educated mothers reported higher perceived efficacy of attaining their possible selves. This suggests that in our study, the educational level of each parent was associated with distinct aspects of possible selves. The role of the parental educational level in the salience of academic possible selves is consistent with previous evidence indicating that the parental SES, which includes educational level, is linked to secondary students’ expectations and aspirations for higher education (Chang et al., 2006; Hadjar et al., 2021; Kerpelman & Mosher, 2004; Schoon & Ng-Knight, 2017; Yau et al., 2021). This finding is also consistent with IBM theory, which posits that salient identities of students from lower-SES backgrounds may not prioritize academic achievement (Oyserman, 2013; Oyserman & Lewis, 2017). Nevertheless, the absence of a notable impact of maternal education on the salience of academic possible selves highlights the necessity for further investigation. The finding that students with more highly educated mothers reported greater perceived efficacy of attaining their possible selves is consistent with previous evidence (Wiederkehr et al., 2015) and underscores the role of the maternal educational background in shaping students’ perceptions of their ability to attain their possible selves.

Students whose parents had a higher educational level reported higher academic achievement, a finding consistent with previous research demonstrating a positive association between academic attainment and the family SES (e.g., Schoon & Ng-Knight, 2017; Wiederkehr et al., 2015). Our findings indicated that having plausible academic possible selves (i.e., academic possible selves linked to strategies), perceiving school as path to the future, or interpreting difficulty in academic tasks as importance are not affected by the parents’ educational levels. This evidence was supported by previous studies (e.g., Carey, 2022; Kerpelman et al., 2008) indicating that parental beliefs and support contribute more than parental educational levels to shaping academic possible selves.

Girls were more likely than boys to report hoped-for and feared possible selves in the academic domain, suggesting that academic aspirations and concerns may be of greater centrality to their self-concept. Additionally, girls reported a higher level of academic possible self plausibility. Our finding that female students are more likely to envision more realistic academic futures is consistent with prior research indicating that girls not only articulate more academic possible selves but also generate more strategies to attain or avoid them (Hadjar et al., 2021; Kerpelman et al., 2008; Kerpelman & Mosher, 2004; Knox et al., 2000; Konidari, 2021; Leondari et al., 2009; Leondari & Gonida, 2008; Oyserman et al., 2011; Yau et al., 2021; Zhu et al., 2014; Zhu & Tse, 2016). Girls also reported greater levels of academic self-regulation and better grades. These findings align with prior research indicating that girls often exhibit better academic outcomes (Horowitz et al., 2020; Schoon & Ng-Knight, 2017).

According to IBM theory, students tend to interpret their experiences and take actions in a gender-congruent way, influenced by situational cues related to gender and academic achievement (Elmore & Oyserman, 2012). Girls may internalize contextual messages that emphasize the congruence between the female gender and education, thereby enhancing their motivational beliefs. This idea is supported by our finding that girls reported higher levels of perceived teachers’ beliefs about school value. These perceptions may reflect the impact of societal expectations and gender norms that promote educational attainment for females. As we did not directly measure teachers’ beliefs but students’ perceptions of them, this finding suggests that teachers may, indeed, have these perceptions about school value or that girls may interpret teachers’ beliefs as being more positive and perceive educational contexts as being more supportive to their efforts.

Regarding differences between school types, the results indicated that general school students were more likely than vocational school students to report hoped-for and feared academic possible selves, a consistent finding with previous research (Koul et al., 2017; Soidet et al., 2022). Additionally, general school students reported higher levels of perception of parental school value beliefs, perception of school as a path, academic possible self plausibility, academic self-regulation, and achievement, as well as lower levels of interpreting difficulty as impossibility compared to vocational school students. No significant differences were found between school types regarding teachers’ beliefs, efficacy for attaining possible selves, or interpreting difficulty as importance. Previous research has also supported that vocational school students often struggle to generate strategies for attaining a hoped-for future and perceive a weaker connection between their current and future selves (Chishima & Wilson, 2021; Konidari, 2024). These findings suggest that the general school context supports students in envisioning plausible academic futures and seeing school as a path for their futures. Vocational school students, on the other hand, may face situational challenges that diminish their academic motivation. The differences in students’ perceptions of their parents’ beliefs about the value of school for their futures suggest the prevalence of differing value systems across school contexts, indicating that different contexts may convey different messages about education.

The above differences support the notion that the contents of possible selves and relevant beliefs are constructed within a specific context, and different educational settings contribute to the development of adolescents’ future self-representations (see Oyserman & James, 2009). Specifically, the characteristics of vocational education may tend to prioritize practical skills over academic aspirations and goals, leading to the perception that further education and academic achievement are incongruent with the “vocational school student identity”. This viewpoint is based on IBM theory, which suggests that identities are situation specific, and people think of themselves and act in ways that are congruent with their salient identities, fitting who they are (Oyserman, 2007).

Another explanation for the differences found between the two school types would be that these may reflect a self-selection process. Specifically, students for whom the academic domain is less salient in their self-concept, those who do not perceive school as a path to their future and believe their parents hold a similar view, and those who have a less adaptive difficulty mindset are more likely to opt for vocational education. This trend can be reinforced among students who demonstrate lower academic outcomes, for whom vocational education may appear as a more suitable option (e.g., Dubeau et al., 2017). Moreover, vocational schools are often populated by adolescents from more socioeconomically disadvantaged backgrounds (Kiprianos & Christodoulou, 2015; Van der Veen & Peetsma, 2019). Our findings also revealed that parental education—a critical aspect of SES—was lower among vocational school students compared to those in general schools. This may prompt these students to perceive academic pathways as being less attainable and experience their educational and social environments as being less supportive of their academic success (Oyserman, 2013; Oyserman & Lewis, 2017), which, in turn, contribute to weaker motivational beliefs and lower academic future orientation within the vocational school population.

### 4.2. Possible Selves and Difficulty Mindsets: The Paths to Academic Self-Regulation and Achievement

In our path model, we examined how perceived parents’ and teachers’ beliefs of school value for their children’s futures, aspects of possible selves, and mindsets about difficulty in academic tasks predicted students’ academic self-regulation and achievement and whether school type (general and vocational schools) moderated the relationships among these variables.

The results of this study indicated a strong model fit for the total sample, as well as within the general and vocational school subgroups. The majority of our hypothetical paths were confirmed, while the school context was found to play a role in some relationships between the study variables. The overall structural model explained a substantial amount of variance in academic self-regulation, particularly among vocational students (60.9%), underscoring the predictive strength of these constructs in this educational context.

The results indicated that perceived parental and teachers’ beliefs were significant predictors of possible selves in both school samples. These findings align with previous research highlighting the role of parents (Carey, 2022; Kerpelman et al., 2008; Oyserman et al., 2007; Zhu et al., 2014) and teachers (Pizzolato, 2006; Van der Veen & Peetsma, 2019) in shaping an academically oriented future. Specifically, perceived parental beliefs positively predicted academic possible self plausibility and the perception of school as a path to possible selves, while perceived teachers’ beliefs positively predicted the perceived efficacy of attaining possible selves. This finding indicates that the influence of significant others on adolescents’ perceptions about themselves and education may vary depending on the specific “messages” they convey regarding the value of education for their children’s or their students’ futures. Parents’ messages may be more important in shaping how students conceptualize their possible selves in the academic domain, whether they employ relevant strategies, and the extent to which they perceive their school and their future as being connected. On the other hand, teachers’ messages are important in shaping students’ efficacy beliefs, regardless of the school they attend.

Notably, among general school students, the academic possible self plausibility was not a significant predictor of either difficulty mindsets or academic outcomes. In contrast, among vocational school students, academic possible self plausibility was found to negatively predict difficulty as impossibility and positively predict academic achievement. The positive association between academic possible self plausibility and students’ academic achievement has been supported in previous studies conducted among low-income and high-poverty students (Horowitz et al., 2020; O’Donnell & Oyserman, 2022; Oyserman et al., 2004). In our study, this significant association was confirmed only for vocational students. This finding suggests that plausible academic possible selves may particularly matter for students in more vulnerable contexts. These results also underscore the importance of cultivating academic possible self plausibility among vocational school students, as this can lower a difficulty-as-impossibility mindset and enhance academic outcomes.

On the other hand, the perceived efficacy for attaining possible selves was identified as a significant predictor only within the general school sample, indirectly predicting academic self-regulation through both difficulty mindsets and academic achievement through difficulty as impossibility. According to IBM theory, perceived efficacy can imply relevance, fostering adaptive interpretations of experienced difficulty and better academic outcomes (Oyserman, 2024; Oyserman & Horowitz, 2023). Our findings align with previous studies that have demonstrated a connection between difficulty as impossibility and constructs related to perceived efficacy. Burbidge et al. (2024) found that higher academic possible self-certainty reduced difficulty as impossibility, while Aelenei et al. (2017) found that difficulty as impossibility was linked to the likelihood of attaining academic possible selves among university and college students. However, in our study, this association was significant only among general school students, possibly because vocational school students’ possible selves are less tied to the academic domain, making perceived efficacy less of a contributing factor in the vocational sample model. This finding warrants further exploration, indicating that the school context matters for these beliefs.

In contrast to possible self plausibility and efficacy, the perception of school as a path was identified as a significant predictor in both school contexts. In both school groups, this perception directly predicted academic self-regulation and achievement, aligning with previous findings (Landau et al., 2017; Nurra & Oyserman, 2018). Moreover, the perception of school as a path indirectly predicted academic outcomes through distinct paths for each school. Among vocational school students, the perception of school as a path indirectly predicted academic self-regulation through the difficulty-as-importance mindset. In contrast, among general school students, the perception of school as a path indirectly predicted academic achievement through the difficulty-as-impossibility mindset (negatively) and academic self-regulation through both difficulty mindsets. These results highlight the perception of school as a path as being a critical factor for shaping both difficulty mindsets and academic outcomes in both school contexts. Moreover, our findings indicated that difficulty as importance and difficulty as impossibility are distinct constructs and differentially contribute to other variables, consistent with what previous studies have found (see Oyserman, 2024).

The multigroup analysis revealed significant differences between the general and vocational school students in specific path coefficients. First, the negative effect of interpreting difficulty as impossibility on academic outcomes was more pronounced among general school students. Specifically, difficulty as impossibility was found to predict school achievement only in general school students, while its effect on self-regulation was stronger in general than in vocational school students. Second, the positive effect of the school as a path perception on difficulty as importance was stronger for vocational than general school students, highlighting the role of this perception in the interpretation of difficulty as importance among vocational students. These differences underscore the significance of the diverse school context in shaping motivational perceptions as, in our case, possible selves and their related variables. Although general school students reported lower levels of difficulty as impossibility compared to vocational school students, this mindset appears to contribute more to the general school sample, indicating that when such a mindset is present, it does have important effects. Additionally, our results indicate that the school context influences whether and which difficulty mindsets matter, contributing to the existing evidence highlighting the role of the educational context. Specifically, community college students—considered as a more academically vulnerable group—benefited more than university students when guided to interpret difficulty as a sign of importance rather than impossibility (Aelenei et al., 2017). Oyserman (2024) also states that students in deprived environments, such as high-poverty contexts, may benefit more from interventions promoting difficulty as importance.

Overall, the findings of the present study indicate that many of the variables predicting motivational beliefs and academic outcomes were similar across general and vocational school contexts. In particular, the perceived relevance of the current education for students’ futures, as reflected in the perceived beliefs of parents and teachers about the value of school and students’ perception of school as a path, emerged as significant predictors in the path model for both school contexts. These results highlight the importance of fostering these perceptions among students, regardless of their school type. However, despite these similarities, some paths differed between the two groups, implying different dynamics in the two contexts. This finding underscores the critical role of the educational context in shaping the relationships between motivational constructs and academic outcomes, consistent with IBM theory, which emphasizes the situational specificity of possible selves and their motivational consequences.

### 4.3. Limitations, Future Directions, and Practical Implications

Our study has several limitations that future studies should address. First, the cross-sectional design, with data collected at a single time point, limits our ability to examine developmental trends or changes in the content and structure of possible selves over time. Moreover, this study cannot provide insights into the causal direction of the relationships between the motivational beliefs explored and academic outcomes (i.e., self-regulation and achievement). Second, this study was conducted within the Greek sociocultural and educational context, which restricts the generalizability of its findings to other countries with different school systems. Moreover, the reliance only on self-reported measures raises concerns about potential biases. Future studies should incorporate various sources, such as teachers’ reports and school records, to measure students’ academic self-regulation and achievement. Another concern is that there was an imbalance in the sample sizes of the two groups (210 vocational school students and 388 general school students), which may have an impact on the results of the total sample model and on the comparisons between the two school settings. Future studies should aim to achieve more balanced group sample sizes. In addition, future research could incorporate qualitative methods, such as interviews or focus groups with students and teachers, to explore their perspectives in greater detail and to gain deeper insights into the findings.

Despite these limitations, our findings indicated significant factors in the possible-self literature predicting students’ academic outcomes, such as self-regulation and achievement during adolescence, which have clear implications for practice. The results highlight the need for targeted interventions for vocational school students, boys, and students with less-educated parents to enhance their motivational beliefs and, in turn, their academic outcomes. Furthermore, our findings underscore the role of parents’ and teachers’ beliefs, emphasizing the importance of educating them to support adolescents in envisioning a future that is more education-oriented and connected to their present choices and their current school contexts. By acknowledging the differences between general and vocational education settings, these findings highlight the importance of tailoring interventions to support diverse student populations. Specifically, vocational school students may particularly benefit from interventions that encourage them to view school as a path to their futures and to develop more plausible academic possible selves, that is, to couple their future self-representations with concrete strategies and actions to attain them. Strengthening the school as a path perception for these students appears to be a more beneficial way toward a more adaptive difficulty mindset which, in turn, predicts academic self-regulation. Conversely, for general school students, efforts to weaken their interpretation of difficulty as impossibility may be more impactful, as this mindset appears to have a stronger association with their academic outcomes. A way to shape this mindset is by helping students to see school as being connected to their futures and perceive themselves as capable of attaining their hoped-for possible selves.

Furthermore, our findings highlight the importance of not only addressing differences between school types but also further examining how teaching practices across both educational contexts can foster future-oriented academic thinking, positive beliefs about the academic self and education, and positive academic outcomes. Regardless of the school type, our results suggest that teaching students the relevance of their education for their academic and career futures, as well as ways to connect current actions with their future aspirations and fears, could benefit both general and vocational school students. Additionally, because these findings underscore the role of both parents’ and teachers’ beliefs, our study emphasizes the importance of equipping them with tools to support adolescents in envisioning an education-oriented future that is closely connected to their present choices and current school contexts. Counseling and educational programs could empower the positive influences of parents and teachers by enhancing their ability to convey the value of education and provide meaningful support.

School and counseling psychologists, as well as educators, can apply the insights from this study to strengthen students’ possible selves and motivational beliefs within the specific contexts of general and vocational schools. This can be achieved through counseling and evidence-based programs designed to promote plausible academic possible selves, link future self-concepts to current academic efforts and efficacy beliefs, and foster adaptive difficulty mindsets.

## Figures and Tables

**Table 1:** Academic possible self plausibility coding system.

Plausibility Score	Count of Hoped-for and Feared APSs	Count of Strategies Attached to APSs	Combination of APS and Connected Strategies
0	0	0	0 APSs and 0 STRs
1	1	0	1 APS and 0 STRs
2	1	1	1 APS and 1 STR
2	2	0	2 APSs and 0 STRs
3	1	≥2	1 APS and ≥2 STRs
3	2	1–2	2 APSs and 1–2 STR(s)
3	3	0–1	3 APSs and 0–1 STR(s)
3	4	0	4 APSs and 0 STRs
4	2	≥3	2 APSs and ≥3 STRs
4	3	2–3	3 APSs and 2–3 STRs
4	4	1	4 APSs and 1 STR
5	3	≥4	3 APSs and ≥4 STRs
5	4	2–3	4 APSs and 2–3 STRs
6	≥4	≥4	4 APSs and ≥4 STRs

Notes: APS = Academic possible self; STR = Strategy.

**Table 2:** Correlations among study variables by school-type group.

Scale	1	2	3	4	5	6	7	8
**General School Students**								
1. Perceived parents’ school value	-							
2. Perceived teachers’ school value	0.253 ***	-						
3. School as a path	0.361 ***	0.183 ***	-					
4. Difficulty as importance	0.138 **	0.176 ***	0.449 ***	-				
5. Difficulty as impossibility	0.004	0.005	−0.193 ***	−0.106 *	-			
6. Perceived PS efficacy	0.074	0.221 ***	0.350 ***	0.304 ***	−0.262 ***	-		
7. AcPS plausibility	0.206 ***	0.070	0.323 ***	0.090	−0.083	0.122 *	-	
8. Academic self-regulation	0.039	0.142 **	0.512 ***	0.542 ***	−0.356 ***	0.273 ***	0.133 *	
9. School performance	0.131 *	0.086	0.347 ***	0.238 ***	−0.388 ***	0.155 **	0.138 **	0.377 ***
**Vocational school students**								
1. Perceived parents’ school value	-							
2. Perceived teachers’ school value	0.469 ***	-						
3. School as a path	0.494 **	0.248 ***	-					
4. Difficulty as importance	0.410 ***	0.163 *	0.639 ***	-				
5. Difficulty as impossibility	0.090	0.018	−0.081	0.031	-			
6. Perceived PS efficacy	0.190 **	0.262 ***	0.319 ***	0.286 ***	−0.125	-		
7. AcPS plausibility	0.214 **	0.120	0.408 ***	0.191 **	−0.176 *	0.146 *	-	
8. Academic self-regulation	0.300 ***	0.130	0.705 ***	0.696 ***	−0.128	0.250 ***	0.302 ***	
9. School performance	0.102	0.079	0.322 ***	0.247 ***	−0.131	0.053	0.286 ***	0.352 ***

* *p* < 0.05; ** *p* < 0.01; *** *p* < 0.001.

**Table 3:** Indirect effects.

Paths for Specific Indirect Effects	*Β*	95% C.I.s for Indirect Effects	
		LB	UB
General school students			
PSV→Spath→ACSR	0.097 **	0.055	0.140
PSV→Spath→Dif_Import→AcSR	0.050 **	0.028	0.072
PSV→Spath→GPA	0.078 ***	0.034	0.122
PSV→Spath→Dif_Impos	−0.040 *	−0.078	−0.002
PSV→Spath→Dif_Import	0.131 ***	0.080	0.181
TSV→PSef→Dif_Import→ACSR	0.014 *	0.003	0.026
TSV→PSef→Dif_Impos →ACSR	0.012 **	0.004	0.021
TSV→PSef→Dif_Impos→GPA	0.016 **	0.005	0.027
TSV→PSef→Dif_Impos	−0.048 **	−0.078	−0.019
TSV→PSef→Dif_Import	0.037 *	0.007	0.068
Spath→Dif_Import→GPA	0.038 *	0.002	0.075
Spath→Dif_Impos→GPA	0.039 *	0.004	0.075
Spath→Dif_Import→AcSR	0.149 ***	0.097	0.201
Spath→Dif_Impos→AcSR	0.031 *	0.003	0.059
PSef→Dif_Import→ACSR	0.066 **	0.026	0.105
PSef→Dif_Impos →ACSR	0.057 ***	0.026	0.088
PSef→Dif_Impos→GPA	0.072 ***	0.032	0.112
Vocational school students			
PSV→Spath→ACSR	0.201 ***	0.127	0.276
PSV→Spath→Dif_Import→AcSR	0.119 ***	0.064	0.173
PSV→Spath→GPA	0.115 *	0.021	0.210
PSV→AcpS→Dif_Impos	−0.039 *	−0.075	−0.003
PSV→Spath→Dif_Import	0.276 ***	0.189	0.363
Spath→Dif_Import→AcSR	0.246 ***	0.157	0.335

Notes: PSV = perceived parental school value for the future; TSV = perceived teachers’ school value for the future; AcPS = plausibility of academic possible selves; Spath = perception of school as a path to the future; PSef = perceived efficacy for attaining possible selves; Dif_impos = interpretation of difficulty in academic tasks as impossibility; Dif_import = interpretation of difficulty in academic tasks as importance; AcSR = academic self-regulation; GPA = school achievement. All the coefficients are standardized; * *p* < 0.05; ** *p* < 0.01; *** *p* < 0.001.

## Data Availability

The data presented in this study are available on request from the corresponding author because of privacy.
